# Ontario’s Experience of Wind Energy Development as Seen through the Lens of Human Health and Environmental Justice

**DOI:** 10.3390/ijerph13070684

**Published:** 2016-07-06

**Authors:** Emmanuel Songsore, Michael Buzzelli

**Affiliations:** Department of Geography, Social Science Centre, Western University, London, ON N6A5C2, Canada; esongsor@uwo.ca

**Keywords:** wind energy, health risk, environmental justice, Ontario, newspapers

## Abstract

The province of Ontario has shown great commitment towards the development of renewable energy and, specifically, wind power. Fuelled by the Green Energy Act (GEA) of 2009, the Province has emerged as Canada’s leader in wind energy development (WED). Nonetheless, Ontario’s WED trajectory is characterized by social conflicts, particularly around environmental health. Utilizing the Social Amplification of Risk Framework, this paper presents an eight-year longitudinal media content analysis conducted to understand the role Ontario’s media may be playing in both reflecting and shaping public perceptions of wind turbine health risks. We find that before and after the GEA, instances of health risk amplification were far greater than attenuations in both quantity and quality. Discourses that amplified turbine health risks often simultaneously highlighted injustices in the WED process, especially after the GEA. Based on these findings, we suggest that Ontario’s media may be amplifying perceptions of wind turbine health risks within the public domain. We conclude with policy recommendations around public engagement for more just WED.

## 1. Introduction

The growth of alternative energy in recent times has been driven by concerns over energy insecurity and sovereignty, climate change and pollution from carbon-based infrastructure. Ontario, Canada, is among the most ambitious jurisdictions pursuing wind energy development (WED) as indicated by rapid growth after the Province’s Green Energy and Green Economy Act of 2009. Since then, developers in Ontario have taken advantage of WED’s cost effectiveness, deployability and low emissions to install 4361 MW of turbines [1]. This is currently about 40% of Canada’s total installed capacity and nearly 10% of Ontario’s energy mix [1]. In contrast to the aforementioned merits, the technology faces some challenges within certain deployment contexts, a few of which include grid integration and variability [2,3] and concerns over wildlife [4].. Despite the promises of the GEA and WED to deliver clean energy and green jobs, development is fraught with conflict across technical, economic, social and, our focus here, health issues.

The issue of wind turbines and human health in particular presents a complicated and multifaceted case of environmental (in)justice. On the one hand, the Ontario government’s justification for WED rests on its health benefits. For example, the province boasts of being the first North American jurisdiction to phase out coal-generated energy with wind power [1]. On the other hand, the technology continues to be perceived as a public health risk, a subject that remains extremely controversial even within the scientific community. This paradox creates multiple understandings of what might constitute “just” WED. 

We recognize the roles played by the media in both reflecting and shaping public perceptions around wind turbines and health [5,6]. Through the lens of the Social Amplification of Risk Framework and newly-emerging theories of energy justice, we conduct a longitudinal media content analysis to: (1) understand how wind energy is portrayed as a health risk or benefit and (2) understand the nature of justice discourses in the context of WED and health. Based on the significant role played by the Green Energy and Green Economy Act (GEA) as a driver of WED, we seek to understand how these discourses have evolved relative to the policy. This research finds that policy could benefit by understanding and incorporating the perspectives of key stakeholders (particularly local communities), often presumed as defiant for opposing projects [7]. We conclude with a discussion of justice-informed policy alternatives that may offer a more consensus-based path to renewable energy development.

## 2. Emerging Literature on (Wind) Energy Justice and Wind Turbine Health Effects

WED and environmental justice (EJ) have only recently begun to appear together in the research literature, yet they offer interesting points of analysis and mutual exchange. Development controversies grow out of a fundamental conundrum in the sector: that generally-accepted and lauded societal goals of renewable and clean energy often run counter to local community concerns. Accordingly, we are presented with fruitful conceptual and empirical research paths [8], including: policy conflicts between environment and health goals and priorities; global versus local priorities within development; the ways in which sustainable development might falter when policy (theory) meets implementation (practice). Community concerns around health issues, we argue, provide a new path through which to define and analyse WED. In the context of this paper, we contextualize health as including turbine impacts on “physical, metal and social wellbeing” [9].

Two major conflicting non-academic publications that have been impactful in shaping discourses around WED in Ontario include “Wind Turbine Sound and Health Effects: An Expert Panel Review” [10] and “Wind Turbine Syndrome: A Report on a Natural Experiment” [11]. While the former was funded jointly by American and Canadian Wind Energy Associations, the latter was conducted by Nina Pierpont, a New York-based physician. These publications have suggested the existence and nonexistence of health impacts, respectively. Despite a range of health concerns around WED, peer-reviewed academic research has pointed to turbines causing annoyance and sleep disturbance associated with proximate turbines’ vibration and noise (“swoosh”) [12,13]. In a systematic review of peer-reviewed literature on turbine health effects, it was concluded that “there is some evidence that exposure to wind turbine noise is associated with increased odds of annoyance and sleep problems” [14], which constitute a direct measure of health [15]. 

Given the environmental health nature of these issues, WED lends itself naturally to analysis from the perspective and priorities of EJ. ‘Process’ EJ studies, those concerned with the histories and policies that generate unjust community outcomes, provide a broad context for us here. Whereas scientific or epidemiologic studies focus on annoyance and sleep disturbance (outcomes), our aim is to understand how health-related EJ tensions arise within the WED process. This conceptualisation of EJ was captured by the U.S. EPA when it referred to just development as “…fair treatment and meaningful involvement of all people…achieved when everyone enjoys the same degree of protection from environmental and health hazards and equal access to the decision making process to have a healthy environment in which to live, learn, and work” [16].

This broad conceptualisation of EJ has been applied to a number of issues and contexts [17], though only very recently, and in particular ways, in the emergent so-called “energy justice” movement [18,19]. Broadly speaking, energy affordability and the politics of infrastructure are the leading equity themes in the energy studies literature [20]. We see these priorities, for instance, in case studies of the historical development of energy infrastructure and energy poverty in North Carolina [21] and in aboriginal land claims against internal colonisation in Sweden [22]. Cowell and colleagues point out that the concepts of fairness and justice used in research on WED have tended to focus on the distribution of economic benefits and/or public engagement in the development process [23]. Thus, we have studies on supply chain impacts of more just energy development [24], what this might mean for ethical energy consumption [25] and the tools that may facilitate the problems imbued in planning in more equitable ways [26]. Present, but never a principal focus of this emergent literature, we argue, is the environmental health lens. Given both its saliency within EJ and its importance to communities within the WED process, we seek to develop this further dimension of energy justice. 

## 3. WED and Health: A Theoretical Frame

Given the contested nature of wind turbine health effects and associated issues of environmental justice that have arisen in Ontario [6,27,28,29], we utilize the Social Amplification of Risk Framework (SARF) to understand how these discourses manifest within media coverage. SARF permits insights into the potential role of media discourse in magnifying or minimizing public perceptions of wind turbine health effects and, in turn, perceptions of justices or injustices in the context of WED. SARF was developed to aid in coherent and integrated understanding of risk perception and communication [30]. The theory posits that “risks, risk events and the characteristics of both become portrayed through various risks signals (e.g., images, signs and symbols) which in turn interact with a wide range of psychological, social and institutional, or cultural processes in ways that intensify or attenuate perceptions of risk and its manageability” [31]. The theory further argues that the experience of risks transcends physical harm and includes the mechanisms through which communities learn about them. Thus, central to this theory is the role played by the mass media in communicating risks to the general public [32].

**Figure 1:**
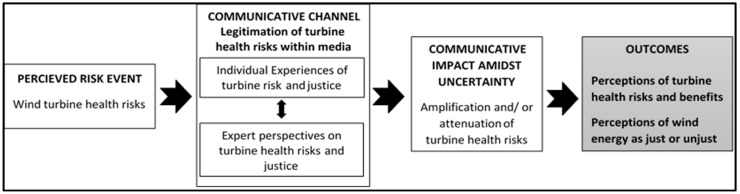
Social Amplification of Risk Framework in the context of wind energy development. Adopted and simplified from Kasperson et al. [30].

Figure 1 provides a conceptual summary of SARF as it relates to the current study. Considering conflicting accounts surrounding wind turbine health effects, we set out to understand how the media acts as a communicative channel that amplifies or attenuates the saliency of turbine health impacts. Specifically, we pay attention to how these health impacts are legitimized through the processes of authorization and normalization. Central to the SARF is the idea that communication from experts usually triggers significant public concerns [30]. Through authorization, we therefore seek to understand how reference to authority within media coverage may act to amplify or attenuate perceptions of turbine health risks [33]. Since turbine health risks remain an uncertainty, our second goal is to understand how exemplarity plays out within the media coverage. With exemplarity, we are interested in understanding how tangible testimonies from individuals and communities living near turbines play out in the media to heighten or minimize wind turbine health effects [34]. Equally importantly, we seek to understand if and how justice- and injustice-based discourses emerge within broader discourses, which tend to amplify and attenuate turbine health risks.

Although the impacts of media discourse on public opinions remains complex and multifaceted, decades of media effects research have identified agenda setting and framing as the main mechanisms through which public discourse and opinions may be impacted by the media. While agenda setting suggests a positive correlation between the amounts of coverage and the importance audiences attribute to issues [35], framing refers to the mode of communication used to present issues [36]. By assessing the quantity (i.e., relative occurrence of amplifications and attenuations) and quality (i.e., mechanisms of legitimation and the nature of justice-based discourses), the current study provides some opportunity for understanding the potential impacts of media coverage on public perceptions. 

## 4. Methods

This section details the methods that were employed in the study. First, we outline the procedures for sampling news articles and their associated rationale, after which the analytical methods (content analysis and thematization) are discussed. 

### 4.1. Newspaper Selection and Sampling

The preliminary sampling procedure involved locating all wind energy projects within Ontario and their respective host communities. Through a search conducted on the website of Ontario’s Ministry of Energy [37], we found 41 projects located in approximately 23 communities. Since we were interested in understanding media coverage within turbine communities, we set out to document all digitally-accessible local newspapers circulated within host communities in order to obtain a large and representative sample of articles. In total, 13 local newspapers circulated in host communities were accessible via the LexisNexis news database. 

**Figure 2:**
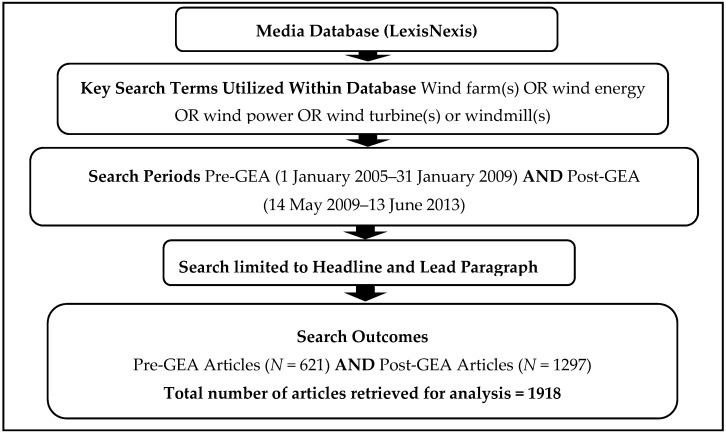
Summary of sampling protocol in LexisNexis. GEA, Green Energy Act.

**Figure 3:**
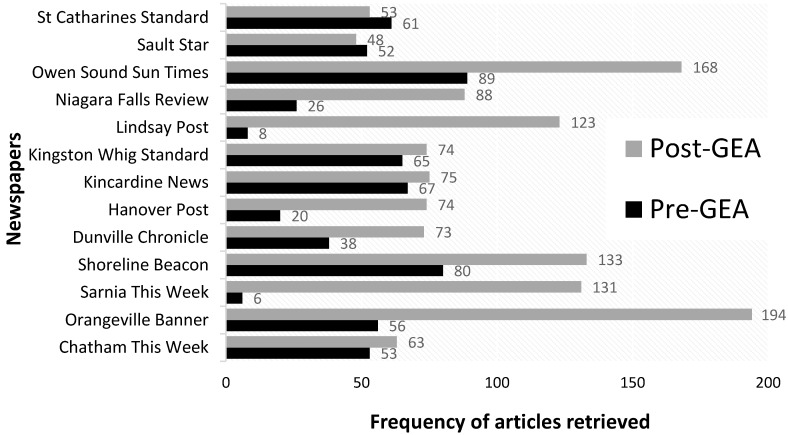
Frequency of sampled articles by newspaper source.

The next step involved selecting a date range for sampling. Prior to 2005, we found several inconsistencies within the database (e.g., missing news articles). We therefore decided to sample articles published between January 2005 and June 2013 (the search month). Since we wanted to understand variations in wind energy health effects and justice discourses relative to Ontario’s GEA, articles were sampled into two broad clusters: both 4 years prior to the policy (1 January 2005–31 January 2009) and 4 years after its implementation (14 May 2009–13 June 2013). Figure 2 summarises the procedures used to retrieve articles from LexisNexis. The total number of articles retrieved before and after the GEA was 621 and 1297, respectively. Within almost all newspaper sources, there was a substantial increase in wind energy coverage following the GEA. Specifically, stories on WED were likely to appear 3 and 6 times a week pre- and post-GEA, respectively, indicating that coverage doubled following the implementation of the policy. The list of newspaper sources and the frequency of articles published within each sources relative to the GEA is displayed in Figure 3. 

### 4.2. Data Analysis

The large newspaper article sample necessitated a method that allows for scrupulous and efficient data reduction and analysis. We therefore utilized content analysis, which in the words of Patton [38], is “a qualitative data reduction and sense making effort that takes a volume of qualitative material and attempts to identify core and consistent meanings”. We also relied on content analysis because the methodology aims for systemization, objectivity and reliability, all of which increase rigor [39,40,41]. Thus, it requires that data be sampled and analyzed using a “step by step protocol” [41] with “explicitly formulated rules and procedures” [42]. 

The analysis was preceded by the development of an analytical codebook, which provided explicit article content coding instructions [43]. Based on the study goals, we had two major categories for the coding, i.e., amplification and attenuation of wind energy health risk. While the former represented claims that wind turbines were a health risk, the latter involved suggestions of the non-existence of health risks or the minimization of health risks associated with wind power [31]. In order to be coded, attenuations and amplifications had to be legitimized through authorization or exemplification. An intercoder reliability test was then performed between two researchers who coded a sample of 50 purposefully-selected articles. Intercoder reliability tests enhance the reliability of coded content by measuring the “extent to which independent judges make the same coding decisions in evaluating the characteristics of messages” [44]. A Scott’s Pi (1 > π > 0) test revealed a satisfactory reliability score of 0.83 [45]. All articles were then imported into NVivo 9 for analysis. A total of 889 instances of health risk amplifications and attenuations in the context of WED were coded.

While the content analysis captured the instances of risk amplification and attenuation, the major themes discussed within news articles had to be documented. We therefore conducted a secondary thematic analysis to identify these common themes and patterns that were evident in amplifications and attenuations, as well as the occurrence of justice- and injustice-based discourses amidst risk coverage [46].

## 5. Results

The results of the study are presented in three broad clusters. We start by briefly discussing the prominence of amplifications and attenuations coded before and after the GEA. In Section 5.1, we report the analysis of the amplifications and attenuations of turbine health risks through the mechanisms of normalization and exemplification. In Section 5.2, we present the occurrence of justice- and injustice-based discourses amidst amplifications and attenuations of wind turbine health effects. What we see through media content is both community anxiety around turbines and perceived health effects, as well as a process-based discourse around the planning and implementation of wind energy; both issues that speak directly to environmental injustice.

**Figure 4:**
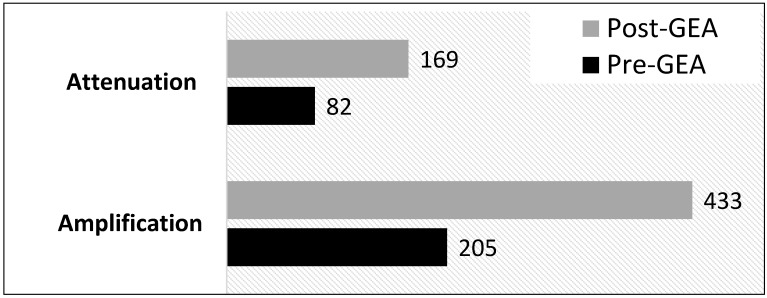
Prominence of reported amplifications and attenuations pre- and post-GEA.

Figure 4 shows the frequency of health risk amplifications and attenuations that were coded before and after the GEA, respectively. The amplification of health risks was substantially higher than instances of risk attenuation both before and after the GEA, respectively. Specifically, instances of amplification were more than twice the frequency of attenuations within both time periods. Based on the media theory of agenda setting, which purports a direct relationship between media coverage and issue saliency [47], public perceptions of wind turbines in the context of health are therefore likely to be more negative than positive. Accordingly, the next section digs deeper into the nature and characteristics of these amplifications and attenuations in order to understand the potential impacts of media coverage on public perceptions.

### 5.1. Amplification and Attenuation of Health-Based Discourse before and after the GEA

Table 1 provides a summary of all of the legitimation mechanisms that were adopted before and after the GEA. Although amplifications and attenuations were evident before and after the GEA, there were major variations in the nature and characteristics of accompanying legitimations. The use of authorization to amplify and attenuate wind turbine health risks generally occurred in two major forms: (1) though statements directly advanced by health experts and (2) through studies conducted by various experts. Exemplification instead occurred via direct statements advanced by individuals who were living within the vicinity of turbines or those who came in close contact with turbines. Pre- and post-GEA discourses pertaining to justice and fairness were predominantly advanced in the contexts where wind turbine health risks were being amplified. In this context, the focus was mainly on the negative treatment of citizens regarding concerns around the negative impacts of turbines. 

#### 5.1.1. Pre- and Post-GEA Attenuation

The attenuation of wind turbine health effects both before and after the GEA took different forms, which included individual testimonies, health expert claims and evidence rooted in scientific research. These attenuations tended to curtail the idea that turbines were harmful by suggesting the nonexistence of negative health effects. Prior to and after the GEA in both time periods, testimonies of individuals tended to suggest that wind turbines were harmless. A majority of these legitimations were carried out by Ontarians who were exposed to turbines over short periods (e.g., individuals visiting and/or touring wind farms). This is exemplified in the following excerpts from news reports, which highlight the perspectives of some Ontario residents before and after the GEA, respectively:

Joyce and Tom Hunter have checked out AIM’s (AIM PowerGen Corporation) 66-turbine wind farm in Norfolk and Elgin Counties, mills on Scottish hills and a wind power plant in Prince Edward Island. Joyce said…they are so quiet you can stand at the base of one of those towers and carry on a normal voice conversation with no problem. The only sound you hear is a swish as the blade passes overhead.

[48]

Nichols says he purposely parked beneath a turbine and found the noise to be minimal and no worse than background noise heard every day. “Certain people just do not want change,” he says. “We do not like coal and we are afraid of nuclear. Do we want to go back to chopping wood?”

[49]

Both of the above quotes demonstrate testimonies of individuals attenuating the health effects of noise from wind turbines before and after the GEA, respectively. Evident in the second quote is another thread that was common within individual attenuations: the comparison of wind turbines to other energy generation, such as coal, which were often described as a greater evil. In another similar instance, a resident who was reported to have claimed that turbines were safer than other energy generation technologies was quoted as posing the following questions “Which is more harmful to you and your children, energy produced from coal burning smokestacks, energy from a nuclear power plant or energy produced from a 400-foot wind tower?” [50]. Also noteworthy is the fact that individual attenuations often acknowledged the existence of turbine noise, an issue that has generated controversy to present day.

In the post-GEA period, there started to emerge reports of occasional attenuations via testimonies of individuals living within the vicinity of turbines. This is captured in the following report about a family who lived within the vicinity of turbines:

The majority of people living near the local turbines don’t appear to suffer the symptoms experienced by some nearby residents. “We’re having no problems at all,” says Melancthon’s Randy Nielsen, speaking for his wife and two teenage children. “We're surrounded by turbines. …We’ve had people come to visit and they all like them.”

[51]

Within the media coverage, there was also evidence of health risk attenuations, which took the form of studies and the direct voices of experts within the scientific community. Regarding the former, expert reports in the Canadian context were used to legitimize the nonexistence of negative health impacts from turbines both before and after the GEA. The Expert Panel Report sponsored by the American and Canadian Wind Energy Associations (AWEA and CANWEA respectively) was often cited. Based on a review of the literature on the health effects of wind turbines, the panel was said to have come to the following conclusions:

In a report released last month, CANWEA states, “Surveys of peer-reviewed scientific literature have consistently found no evidence linking wind turbines to human health concerns. It is important to note that all wind energy projects are required to undertake environmental assessments that assess the potential impacts of wind turbines on ecosystems and human health...”

[52]

Similarly, before the implementation of the GEA, the Natural Resource Department of Canada was also said to have released a report, which “found no problems with low-frequency noise, also known as infrasound” with measurements indicating that “sound at infrasonic frequencies below typical thresholds of perception; infrasound is not an issue” [53]. Unique to post-GEA media coverage, the attenuation of health risks was further intensified through the citing of studies conducted by international experts. For example, the Massachusetts Department of Environment Protection was said to have released a report that found “no scientific evidence to support most claims about ‘Wind Turbine Syndrome’, infrasound effects and harm blamed on wind power such as pain and stiffness, diabetes, high blood pressure, tinnitus, hearing impairment, cardiovascular disease and headache/migraine” [54].

A final strategy that was evident in the attenuation of turbine health risks was explicit voices of various health experts within Ontario. Prior to the GEA being implemented, a major voice that was dominant in all the newspaper sources was that of David Colby, the health officer of Chatham-Kent, Ontario. This quote captures the broad range of claims that were advanced by Colby:

Chatham-Kent’s acting health officer is reiterating his position that wind turbines planned for the community pose no real health threats...Colby cited turbine failure, icing in northern climates, sound emissions and noise concerns, shadow flicker and construction injuries as the key areas of concern….He also says the noise produced by the wind turbines should not cause a problem to humans. Colby also states that shadow flicker, which some believe could trigger epileptic symptoms in susceptible people, is well below the threshold at which that would happen.…Colby states that “... opposition to wind farms on the basis of potential adverse health consequences is not justified by the evidence.”

[55]

Similar claims by Colby continued to emerge after the GEA. For example, on one occasion, it was reported that while Colby did not claim to be an expert on the subject of wind turbine health effects, his conclusion about the nonexistence of a scientific link was based on a review of “existing scientific reports and medical journals” [56]. Similar claims were reportedly made by Warren Mabee, a professor and renewable energy researcher at Queens University who was quoted as saying “there have been studies that have been done in Canada and elsewhere. None of them have come back conclusively saying that wind turbines are the cause of this type of health issue” [57]. Following the GEA, it was reported that Loren Knopper, an environment and health consultant in Ontario, stated that around the world, government agencies have found that, properly cited turbines do not impact human health negatively [58].

Similar to individuals, the attenuation of wind turbine health effects by Canadian-based experts occasionally involved comparing the relative risks associated with other energy generation technologies to wind power. This was evident only after the GEA was passed into law and is exemplified in an excerpt from an article that highlighted potential health benefits of wind power:

On the other hand, we know that using fossil fuels for energy has profound effects on human health—and on the economy. The Canadian Medical Association reports that in 2008 air pollution in Canada was responsible for 21,000 premature deaths, 92,000 emergency room visits and 620,000 visits to a doctor s office.

[59]

#### 5.1.2. Pre- and Post-GEA Amplification

The amplification of wind turbine health risks took very diverse forms, including testimonies from individuals, expert perspectives (both local and international) and a broad range of studies conducted in different jurisdictional contexts. Unlike the attenuation of wind turbine health effects by individuals who often had sporadic experiences with turbines, testimonies amplifying health risks before and after the GEA were almost exclusively advanced by individuals who lived within the vicinity of turbines. Health impacts reported pre- and post-GEA included sleep deprivation, headaches, nausea, heart palpitations, ear problems, breathing impairments, dizziness, migraines and a host of others. It was often reported that, based on these impacts, some families had to abandon their homes. These testimonies were documented both within and outside Ontario. After the GEA, a resident of Kincardine, Ontario, was for instance reported to have been suffering negative turbine health effects as follows:

Norma Schmidt, who lives in the midst of 110 turbines in Kincardine, said she’s suffered severe migraines, sleep deprivation, weight loss, dizziness, and nauseousness as well as cognitive impairment and ear pressure in the past 22 months. She says there are 14 wind turbines within a two-mile radius of her home west of Underwood. “I have three to five migraines a week and those just continue to increase with the introduction of more turbines…”

[60]

Another common story reported repeatedly prior to the GEA was that of a family who lived in the province of Nova Scotia, Canada. This family’s experience is captured in the following excerpt:

The d’Entremonts literally fled the home they had built in Pubnico Point…a year after a wind turbine was installed 1000 feet away, one of 17 within 1.6 km of their home. During that period, the d’Entremonts say they and their six children suffered a variety of afflictions ….From sleep disruptions to vision and skin problems and increased aggressiveness in several of their children, the d’Entremonts believe the maladies were caused by an acute sensitivity to the vibrations produced by the turbine. They say the majority of the ailments disappeared once the family left their home.

[61]

It is noteworthy that this turbine setback far exceeds the 550-m standard in Ontario. Similar to the above quote, several individual-based amplifications of wind turbine health risks stressed the negative impacts turbines were having on children. For example, a family living in the township of Melancthon, Ontario, were reported to have claimed that “with four small children, two under the age of four, this noise has made for many sleepless nights” [62], while in another article, it was reported that “little children are now experiencing terrible earaches and headaches that they didn’t have prior to the start-up of the wind farm” [63].

Similar to risk attenuation, the amplification of health risks before and after the GEA was dominated by the voices of local experts in Ontario and Canada. However, pre- and post-GEA, the amplification of health risks via the voices of international-based health experts was significantly more pronounced. This trend was even more evident after the policy was passed into law. For example, post-GEA amplifications were dominated by expert voices drawn from all over Europe, the United States and Australia in particular. Two dominant expert voices evident in these amplifications were Nina Pierpont, a New York-based physician, and Robert McMurtry, a former dean of medicine at Western University in Ontario. Pierpont’s concept of wind turbine syndrome is captured here:

Pierpont followed families living within 2 km of wind turbines and describes symptoms such as difficulties with concentration, balance, headaches, nausea, dizziness, irritability, fast heart rate, feelings of panic and ringing in the ears. People most likely to be affected include those prone to migraines, motion sensitivity, inner ear damage as well as extremes of age. As a specialist in behavioral pediatrics, she also expressed concerns with children’s attention, cognition and ability to learn when living in close proximity to wind turbines.

[64]

Once again, we read about health concerns at distances that exceed Ontario’s current setback standard. In a different article, McMurtry was reported to have claimed that some individuals were leaving their homes due to negative impacts from turbines, while others were developing hypertensive symptoms. Stressing the fact that turbines were a problem, he went on to claim that “all the victims have one thing in common. When they go back home, or near the wind farms, they're worse and when they get away, they're better” [65]. 

In comparison to risk attenuation pre- and post-GEA, reported studies that tended to amplify wind turbine health risks within the news articles were far more diverse. For example, studies in France, Denmark, New Zealand and the United States were used to amplify turbine health risks. Following the GEA’s implementation, a study conducted by Jeffery Armini, a health consultant in Guelph, Ontario, Christopher Hanning from Leicester University’s hospital in the U.K. and Michael Nissenbaum, a medical doctor from Fort Kent in the United States, was cited as follows:

we conclude that the noise emissions of iWts (industrial wind turbines) disturbed the sleep and caused daytime sleepiness and impaired mental health in residents living within 1.4 km of the two iWt installations studied. Industrial wind turbine noise is a further source of environmental noise, with the potential to harm human health

[66]

Prior to the policy, a collection of studies by different experts was also cited as follows:

the European Society of Cardiology issued in November 2005, a press release stating “Major study links chronic noise exposure to risk of heart attacks.” In March 2006, the French Academy of Medicine, at the request of their Ministry of Health, issued a report which in translation states, “true risks of the operation of the wind mills are related to the possibility of a chronic sound traumatism, whose physiopathological parameters are well known…”

[67]

Based on the results presented, the amplification of turbine health risks was generally more profound in comparison to risk attenuations. At the individual level, for instance, a majority of attenuations were advanced by people who claimed to have visited wind farms, while amplifications were often advanced by individuals who lived close to wind farms reporting various symptoms and often claiming that they were forced to leave their homes. These experiences together rendered amplifications more tangible in comparison to attenuations. Individual discourses that attenuated wind turbine health risks often acknowledged some controversial characteristics of the technology, such as sounds and noises. On the other hand, individual amplifications predominantly placed explicit emphasis on negative health impacts associated with the technology. Finally, individual attenuations were generally Ontario-based, while amplifications contained testimonies within and outside Ontario. This paints the picture that negative health impacts of turbines are more widespread.

Similar to individual testimonies, the amplification of wind turbine health risks by experts (i.e., scientific voices and studies) painted a more acute picture than risk attenuations. Expert voices and studies that tended to attenuate health risks were mainly Canadian and specifically Ontario-based, with the exception of the post-GEA period, which included non-Ontario-based experts being cited. The credibility of these local sources that tended to attenuate turbine health risks tended to be questioned within the media coverage. For example, although the joint CANWEA-AWEA expert panel review tended to attenuate turbine health effects within pre- and post-GEA coverage, it was often questioned within news reports. In one case, it was stated that the report was produced by a “panel picked and paid for by the wind industry” and further stated that “It’s not a health study. What it’s doing is reviewing the literature. It says over and over again what's needed is further studies” [68]. Contrary to this trend, amplifications were generally rooted in voices and studies by experts in Ontario and abroad. These sources together painted the picture that the negative health impacts of turbines are a global phenomenon. Additionally, most of the impacts reported by these experts were in contexts where wind turbine setbacks far surpassed those established by the government of Ontario. In a jurisdictional context that is relatively new to WED (i.e., Ontario), the media contents analysed tend to present a more holistic and profound picture that supports the existence of health risks associated with wind turbines. 

### 5.2. Justice and Fairness amidst Health Risk Amplification and Attenuation

Within the media contents analysed, justice-based discourses were interwoven with broader discourses amplifying wind turbine health effect before and after the GEA, respectively. These discourses together highlighted procedural injustices pertaining to WED and health in Ontario. These procedural issues had a number of foci: the lack of public participation in turbine deployment decisions; Ontario’s neglect of community health concerns; Ontario’s prioritization of wind energy business over human wellbeing; the lack of municipal planning control in turbine decisions and unfair siting of turbines in ways that compromise the health of Ontarians. The main variation between these injustice-based discourses pre- and post-GEA was that they became more intense and directed specifically at the GEA after the policy was passed into law. 

Prior to the implementation of the GEA, major procedural injustices that were discussed amidst the amplification of wind turbine health risks included the perception that wind turbines were being forced on communities. As well, there were several suggestions that host communities were being taken for granted and treated as experimental subjects. For example, in one instance, it was stated that “a very real danger exists that, in the haste to embrace clean technology, legitimate concerns about noise are being brushed aside” [69]. Other occurrences in this context are demonstrated in the following quotes:

The rapid development of these farms across our province is alarming. We are all guinea pigs …..We need to look long and hard at concerns reported by homeowners who have been forced to live near wind farms. There are limitless sources of testimonials reporting serious health problems….noise intrusion, vibration intrusion as well as a constellation of other symptoms experienced by many who live near the wind turbines.

[70]

Wind turbines are today’s version of a threatening monster being jammed down the throats of neighbours and localities….Turbines erode freedom of the human mind hour after hour, night after day, virtually forever, like a cellphone ringing incessantly that no one can turn off. To many people, this intrusion into their physical and physiological space has a mental effect analogous to the physical effects of a heavy smoker sitting next to you essentially for life.

[71]

Another major discourse of injustice advanced prior to the GEA was the idea that Ontarians did not have a voice in WED. For instance, Harrington and Fraser, members of Wind Concerns Ontario, were reported to have stated that “we are contacted on a daily basis by people in rural Ontario who are living near wind instillations who are suffering greatly. They feel that they have no voice….setbacks aren’t far enough—that’s the whole thing” [52]. In conjunction with these aforementioned injustice-based discourses pre-GEA, a number of lawsuits by residents living within host communities and calls for moratoriums by various municipalities were reported. 

The period after the GEA was enacted saw more intense discourses pertaining to the perceived unjust nature of WED amidst the amplification of wind turbine health risks. These discourses of injustice tended to speak more directly to Ontario’s GEA. Similar to pre-GEA reports, these injustice-based discourses highlighted the fact that Ontarians were being taken for granted, experimented with and ignored in discussions around wind turbine health impacts. A unique recurring theme within these injustice-based discourses amidst the amplification of health risks was the disabling of municipal powers to take control of planning and siting decisions pertaining to wind power, a key feature of the GEA. The following quote demonstrates the amplification of turbine health risks by a resident of Orangeville, Ontario, interlaced with discourses of injustice:

Ashbee, has had firsthand knowledge of the negative effects after having had to abandon her house, uproot her family and relocate.... after being forced out by excessive noise, low frequency vibration, and electrical problems. She agreed…..that there is nothing in the Green Energy Act that offers any protection for the health of the residents. Ashbee noted there are more than 100 known and reported cases of the detrimental health effects in the province, and not one of the families are being taken care of. “Little children are now experiencing terrible earaches and headaches that they didn’t have prior to the start-up of the wind farm,” she said. She said that these residents are “genuinely suffering” and the government is well aware, and will not work to alleviate the problems.

[63]

In a similar reported instance, a resident who was mounting a legal battle against Suncor Energy (a wind energy company in Ontario) based on health problems being experienced by her and her family stressed that plans to install wind turbines were “nothing but evil”. Describing these injustices, she stated that “we are refugees of the Green Energy Act” [72]. A member of a family in a similar situation (Nicholls) was also reported to have stated that “the failed Green Energy Act of this Liberal government continues to punish Ontarians” [73]. Another key recurrent theme within these injustice-based discourses is the opinion that the province of Ontario was not prioritizing the health of citizens. This is further illustrated in the following quote from the Mayor of Amaranth, Ontario:

Amaranth Mayor Don MacIver certainly didn’t welcome news of the Whittington Wind Project's approval…“The province doesn't seem to want to listen,” MacIver said. “Where is the priority in this government? Pieces of metal or people?”….With residents in his municipality living near turbines already reportedly experiencing adverse health effects, MacIver is wary of even larger ones…. “We’re having problems at 1.5 MW,” he said. “Now, we’re watching the bigger ones come in.”

[74]

Within the context of health risk amplification post-GEA, various regulations, such as wind turbine setbacks, were challenged and claimed to be unfair. Calls for more sufficient setbacks and regulations were backed by regulations from other jurisdictions with greater setbacks, as well as perspectives of various health experts and evidence from research studies. This is exemplified in the following excerpt from a news article:

Across Ontario there are complaints of turbine noise causing annoyance, sleep disturbance and consequent health problems. Medical and other authorities recommend setbacks from homes of 1.5 to 2 km. This advice has been ignored by the wind energy industry and the Ontario government.

[75]

The above quote challenges setbacks for WED in Ontario, which were set at 550 m away from the nearest homes by appealing to the claims of medical professionals. Similar to the pre-GEA era, these news reports amplifying wind turbine health risks and issues of injustices often reported law suits and calls for moratoriums by municipalities and other stakeholder groups across the province. Some municipalities were reported to have passed bylaws that were more protective than those established under the GEA. For example, it was reported in one instance that “38,000 farmers across Ontario just called on the Liberal government for a moratorium on new wind turbine start-ups until health issues are resolved” [76]. Another instance is shown in the following quote:

Plympton-Wyoming council was concerned about that distance, saying there are reports of people becoming ill from the sounds and shadow flicker so close to the turbines. It passed its own bylaw under the Ontario Municipal Act to have the turbines two kilometres away from homes. Mayor Lonny Napper says the bylaw was passed to protect residents’ health —which is a duty of politicians under the act.

[77]

In the quote above, the council in Plympton-Wyoming expressed distrust in setbacks established under the GEA, claiming that the health of communities were still being impacted negatively. Hence, to protect their citizens, they established more stringent setbacks of two kilometres in opposition to setbacks of 550 m established under the GEA.

## 6. Discussion 

Utilizing SARF, the current study conducts an eight-year longitudinal media content analysis to understand the dynamics that underlie the amplification and attenuation of wind turbine health risks within Ontario-based media coverage. The analysis is conducted relative to Ontario’s GEA, a landmark policy change that was aimed at accelerating WED in the province. Further, the study digs deeper into the nature of process justice-based discourses as they occur simultaneously with the amplification and attenuation of wind turbine health effects. 

Before summarising the results and discussing the policy and research contributions, we should acknowledge the limitations of the methods used. First, the study relies on local print and online newspapers to discern the dominant forms of information reaching Ontarians in existing and potential wind turbine host communities. Other forms of media would augment and further texture the picture presented here and could include: radio, Internet blogs, Facebook, resistance group websites and television. Additionally, we acknowledge that not all members of these communities may be reading these newspapers. Hence, the issues discussed in the paper may not apply homogeneously across these localities. Despite using content analysis to ensure rigor and reliability in the coding of news articles, the fact that news articles were coded by the primary researcher could have resulted in some oversights and minor errors. Nevertheless, as a key source of information, local newspapers still mirror and mold community sensibilities around WED and offer insights both for health, EJ research and policy.

In the current study, we find that the amplification of wind turbine health risks far exceeded risk attenuations before and after the GEA, respectively. Additionally, relative to attenuated health risks in the media, the framing of amplified health risks painted a more holistic picture concerning the existence of wind turbine health effects. Based on the agenda setting theory, these characteristics of the quantity and quality of the media coverage together suggest that the media in Ontario is playing a major role in advancing negative perceptions about the health effects of wind turbines among Ontarians. It is also noteworthy that the amplification of turbine health risk became more profound following the enactment of the GEA. The current study provides support for a study by Deignan et al. [78], who, through a study of fright factors in the communication of wind turbine health effects in Ontario’s media, concluded that Ontario newspapers may heighten anxiety and fear among readers.

This study builds upon research in Ontario that points to health and its social mediation as a particularly salient issue and source of conflict in WED [27,29,79]. According to the website of Wind Concerns Ontario (WCO), which acts as an umbrella for a number of citizen groups, the province is currently home to approximately 48 local concerned citizen groups. As many as 70 municipalities have also declared their unwillingness to host wind energy projects [80]. A major driver for these concerns has been health. For instance, among a wide array of issues triggering social conflicts in the context of WED, the only concern explicitly featured on the homepage of WCO’s website is health [71]. Amidst contentions about wind turbine health effects even within the scientific community, this paper shows that local newspapers in Ontario are likely acting as a driver of negative perceptions about the potential impacts of turbines on public health. Through the mechanisms of authorization and exemplification, the current study reveals that Ontario’s local media continuously tells two dominant stories about wind energy and health: (1) the fact that scientific experts all over the world attribute a broad array of negative health impacts to turbines and (2) the idea that individuals all over the world are, in fact, stuffing from the negative health effects caused by turbines. These aforementioned stories likely provide some explanation for the contentious nature of wind turbine health effects, which continues to be witnessed within the province. 

Through an analytical focus on the occurrence of justice-based discourses amidst the amplification and attenuation of wind turbine health effects, we find that such discourses occur often amidst risk-based discourses. Specifically, these discourses highlight procedural injustices in the wind energy development process and governments’ handling of public concerns. As shown here, discourses of injustice emerged prior to the GEA. However, by attempting to streamline the development process through disabling municipal-level power over planning decisions [28], the policy triggered more radical injustice-based discourses. Thus, the media discourse analysed reveals that the GEA acted as a confounder of already existing concerns of injustice. While studies in Ontario have suggested that perceptions of injustice act as a trigger of social conflicts in the context of WED [6,28], this study further highlights that these injustices are likely tied to broader issues. Specific to this study, we find that injustices that have emerged in Ontario are likely rooted in broader perceived health concerns.

How do these research findings speak to wider questions of energy/environmental justice and related research paths going forward? We can answer this first with respect to Ontario’s current approach, which may nonetheless hold lessons for other jurisdiction. For instance, Ontario has replaced its feed-in tariff program that accompanied the GEA with the Large Renewable Procurement program (LRP) [28]. Among the goals of this program are the quest to involve municipalities and ensure that developers engage communities more meaningfully in the development process. Thus, the LRP programs aims in part to address issues of injustice in Ontario’s WED process. The LRP creates a competitive points systems for companies that apply for projects. Among other criteria, developers may earn points for demonstrating quality community engagement prior to being offered contracts. While this will likely promote better community engagement, as has been witnessed so far, it does not address the complexities that emerge within this study. We suggest that the complexity of mixed reports on wind turbine health effects within the media alone seems adequate enough to trigger psychosocial stress and uncertainty among host or potential host communities. To date, there are no mechanisms in place to address concerns and/or coping mechanisms within communities hosting projects. Regarding the policy process, communities could benefit from ongoing engagement with various scientific experts and government officials based on emerging concerns around health. Such a process may minimize perceptions of injustices that stem from communities feeling their concerns are being ignored. As well, it could help address actual health impacts that may be felt within host communities. Another issue that may arise with the LRP is that, in cases where communities are reluctant to embracing projects, developers may decide to pursue other points to the detriment of community engagement.

More generally, we can answer the above question by reflecting on some of the fundamental issues that may impede societal transitions toward renewables or indeed be overrun in ways that citizens and communities broadly deem unjust. This may manifest in myriad ways: via high-level policy conflict between progressive energy development and health policy; global energy development goals versus local concerns around implementation; and relatedly, between policy goals and policy processes. If health is an important theme in the nascent energy justice movement, then development will benefit from meaningful inclusion of community health concerns. Sitting as we do on the cusp of significant transitions toward new energy infrastructures, we suggest that more just transitions can be made “right” more easily now than once investments are put in place.

As we consider alternative avenues forward, further research can build upon the empirical insights of this paper. First, the broad range of themes that occur within the media contents analysed could act as material for designing questionnaires and/or interview instruments for further exploration of community-level risk perceptions pertaining to WED. Future studies of this sort could also utilize alternative media sources, such as resistance group websites, blogs and social media sites, to provide complementary insights on the potential impacts of media discourse on public perceptions of wind turbine health effects and issues of justice. Finally, studies on the construction of news pertaining to environment and health remain nascent (e.g., [81]). Future research on journalistic practices that result in the amplification and attenuation of wind turbine health effects could provide insights on imbalances in media coverage and provide recommendations for more balanced reporting.

Sørensen [82] has drawn attention to the need for technological policy discourse that transcends the themes of “deployment” and “innovation”, acknowledging the major role played by the media as a socialisation agent in the context of WED. It is noteworthy that the impact of media discourse on energy transition has not only been limited to wind power. For example, Skjølsvold [83] in finding that the media presents varying perspectives on bioenergy, suggested that the media may be indeed impacting public perceptions. As well, smart grid media discourse in Irish print media has been found to be problematic by failing to pay attention to crucial issues, such as ownership and scale [84]. The current paper contributes to this broader emerging literature by highlighting the role media discourse may be having on public perceptions of wind power. 

## 7. Conclusions

Local newspapers circulated within Ontario have tended to be dominated by the amplification of health risks when reporting on WED and health. These amplifications are often backed by discourses highlighting injustices in the development process. The findings of the current study correspond with the emergence of concerned citizen groups battling wind power within Ontario based on health concerns and perceptions of injustice. We speculate that among Ontario’s populations who rely on local newspapers for information in WED, discourses around health effects and justice are likely to be dominated by negative perspectives. Studies of risk communication in the context of health have often revealed that the media play a significant role in shaping public perceptions. For example, in a study of the media and genetically-modified foods, Frewer et al. [85] found that “perceptions of negative reporting was associated with higher perceived risks” within the public sphere. The current study thus paves a way to understanding the role Ontario’s media might be playing in shaping public perceptions of wind turbine health effects and, consequently, issues of justice. 

## Figures and Tables

**Table 1:** Legitimation of wind turbine health effects.

Time Period	Individual Experiences	Scientific Evidence		Justice/Injustice Discourse
		Studies	Health Expert Voices	
**Pre-GEA** Attenuation	√	√	√	√
**Pre-GEA** Amplification	√	√	√	×
**Post-GEA** Attenuation	√	√	√	√
**Post-GEA** Amplification	√	√	√	×
